# Polyethyleneimine-Directed In Situ Gold Deposition on Gallium Nitride Nanoparticles for Enhanced Electrochemical Detection of Erythromycin

**DOI:** 10.3390/ijms27062728

**Published:** 2026-03-17

**Authors:** Oana Elena Carp, Denisse-Iulia Bostiog, Elena Laura Ursu, Rares-Georgian Mocanu, Narcisa Laura Marangoci, Ion Tiginyanu, Alexandru Rotaru

**Affiliations:** 1 Centre of Advanced Research in Bionanoconjugates and Biopolymers, “Petru Poni” Institute of Macromolecular Chemistry of the Romanian Academy, Grigore Ghica Voda 41A Alley, 700487 Iasi, Romania; rachita.oana@icmpp.ro (O.E.C.); bostiog.denisse@icmpp.ro (D.-I.B.); ursu.laura@icmpp.ro (E.L.U.); mocanu.rares@icmpp.ro (R.-G.M.); nmarangoci@icmpp.ro (N.L.M.); ion.tighineanu@cnstm.utm.md (I.T.); 2 National Center for Materials Study and Testing, Technical University of Moldova, 2004 Chisinau, Moldova; 3 Academy of Sciences of Moldova, 2001 Chisinau, Moldova

**Keywords:** gallium nitride, gold nanoparticles, polyethyleneimine, erythromycin, differential pulse voltammetry, electrochemical sensing

## Abstract

Hybrid nanomaterials that integrate surface functionality, colloidal stability, and efficient electron-transfer pathways are highly attractive for improving electrochemical sensing performance. Herein, we report the fabrication and evaluation of polyethyleneimine-functionalized gallium nitride nanoparticles (GaN) decorated with gold nanoparticles (GaN-PEI-Au) as a tunable electrode modifier for enhanced differential pulse voltammetry (DPV) detection of erythromycin. Branched polyethyleneimine was employed as a multifunctional interfacial layer to stabilize GaN dispersions, introduce amine-rich surface chemistry, and enable in situ gold nanoparticle formation at the GaN-PEI. The optimized GaN-PEI-Au material exhibited high colloidal stability, a characteristic Au localized surface plasmon resonance in the ~520–525 nm range, and well-defined Au nanoparticles attached to the GaN surface. When applied as an electrode coating, GaN-PEI-Au significantly enhanced the erythromycin oxidation response compared to bare Au and GaN-PEI interfaces, consistent with synergistic increases in electroactive surface area and interfacial charge-transfer efficiency. Under optimized DPV conditions, GaN-PEI-Au-modified electrodes enabled quantitative erythromycin determination with a linear range of 5 nM–2 µM (R^2^ = 0.990), sensitivity of 1.32 × 10^−3^ µA nM^−1^, and a limit of detection of 52.5 nM, while maintaining stable baseline behavior during repeated scans. The reported GaN-PEI-Au nanocomposites represent a robust platform for sensitive electrochemical detection of pharmaceutical compounds.

## 1. Introduction

Electrochemical sensing continues to expand as a practical analytical approach because it can deliver rapid, low-cost measurements using compact instrumentation and scalable electrode formats (e.g., screen-printed electrodes) [1,2]. The best strategy for the improvement of the performance of such systems is interfacial engineering: modifying the electrode surface with nanostructured materials to increase electroactive area, improve charge-transfer kinetics, and stabilize signal output [3]. Recent reports highlight that rationally designed nanomaterial coatings, particularly hybrid architectures that combine complementary functions (conductivity, surface chemistry, and structural robustness), are among the most effective routes for improving the sensitivity and repeatability in electrochemical readout [4,5].

Since gold nanoparticles (AuNPs) are widely used as electrosensing enhancers [6,7,8] because they provide highly conductive nano-interfaces and abundant active sites, they are also well suited to polymer-directed assembly and immobilization on various supports. Polymer-based AuNPs synthesis is also attractive due to the fact that polymers can regulate nucleation and growth and limit uncontrolled aggregation, producing stable and reproducible nanoparticles [9,10]. In particular, for branched polyethyleneimine (PEI), the amine-rich structure can coordinate Au(III) and promote stable PEI-Au interfaces; depending on reaction conditions, PEI has been reported to function both as a stabilizing ligand and as a reducing/formation-promoting component during AuNP synthesis [11,12].

Within the context of modifying the electrode surface with nanostructured materials, gallium nitride (GaN) is an attractive, yet still comparatively underutilized, material for electrochemical sensing [13,14]. GaN-based materials offer high chemical stability and promising electronic properties [15,16], and GaN-containing hybrids have demonstrated strong analytical potential across various target analytes [17]. For example, Pd-Pt nanocomposites on porous GaN have enabled highly sensitive nitrite detection, demonstrating GaN’s effectiveness as an electrocatalytic scaffold [18]. Likewise, Au-decorated porous GaN has been successfully applied for non-enzymatic hydrogen peroxide sensing, showing strong electrocatalytic activity and excellent stability [19]. In addition, GaN nanoparticle-polymer hybrid composites have been reported as efficient electrochemical sensors, supporting the feasibility of GaN-based sensing interfaces beyond traditional carbon- and metal oxide-based systems [17]. Despite these promising reports, GaN remains less explored than conventional carbon nanomaterials or metal oxides for practical sensor coatings, leaving substantial room for GaN-based hybrid designs that combine stable dispersion, controllable nanostructure assembly, and efficient electron-transfer pathways.

Herein, we report the fabrication and evaluation of PEI-functionalized GaN nanoparticles decorated with gold nanoparticles (GaN-PEI-Au). The proposed GaN-PEI-Au sensing interface is particularly relevant for the detection of pharmaceutical contaminants [20,21]. Antibiotics, such as erythromycin, are increasingly recognized as persistent environmental pollutants due to their incomplete removal during wastewater treatment and their contribution to antimicrobial resistance [22,23]. Consequently, sensitive, rapid, and portable analytical tools are urgently needed to complement conventional laboratory-based techniques [22,24]. The GaN-PEI-Au-modified electrodes presented here address this need by enabling differential pulse voltammetric (DPV) determination of erythromycin, highlighting the potential of this hybrid platform for monitoring antibiotics and related contaminants in environmental and analytical applications. In the presented GaN-PEI-Au hybrid interface design, PEI is used to functionalize and stabilize GaN nanoparticles and to create an amine-rich interphase that promotes in situ Au nanodeposition on the GaN-PEI interface, yielding GaN-PEI-Au nanocomposites with tunable gold loading. We then correlate colloidal/optical characteristics and morphology with electrochemical behavior and demonstrate the resulting material as an electrode modifier for DPV detection using erythromycin as a representative analyte. The importance of the present study is therefore the materials concept of a modular GaN-supported, polymer-directed Au nanoarchitecture, intended as a general route toward stable, high-response electrochemical sensing interfaces.

## 2. Results and Discussion

### 2.1. PEI-Mediated Interfacial Formation of GaN-PEI-Au Nanocomposites

**Figure 1:**
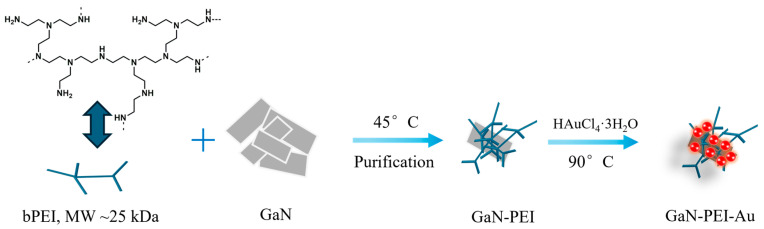
Schematic illustration of PEI-assisted dispersion and surface functionalization of GaN nanoparticles, followed by in situ PEI-mediated nucleation and growth of Au nanoparticles on the GaN surface, yielding the GaN-PEI-Au hybrid nanocomposites.

In our previous work [25], branched polyethyleneimine (PEI, MW ≈ 25 kDa) was shown to be an effective stabilizing and functionalizing agent for gold nanoparticles due to its high density of amine groups capable of coordinating Au(III) and forming robust polymer-metal interfaces. Building on this concept, PEI was used in the current project to disperse and functionalize gallium nitride (GaN) nanoparticles and to create an amine-rich interphase that promotes the in situ formation of Au nanoparticles (AuNPs) on the GaN surface (Figure 1).

Dispersing GaN in a 1% (*w*/*v*) PEI solution, with a subsequent removal of the PEI excess, produced a PEI-associated stable colloidal system in which surface-accessible amines can bind Au(III) precursor species, increasing their local concentration at the GaN-PEI interface and favoring heterogeneous nucleation. Under the applied thermal conditions, PEI is expected to act primarily as a complexing and capping agent and facilitate Au(III) conversion to Au(0), yielding GaN-PEI-Au nanocomposites.

### 2.2. Physicochemical Characterization of GaN-PEI-Au Nanocomposites

#### 2.2.1. Colloidal Properties as a Function of Au Precursor Loading

**Figure 2:**
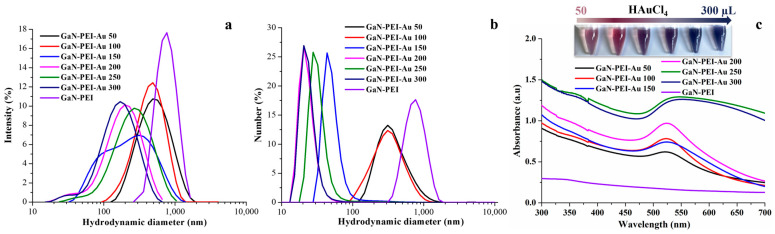
(**a**) Intensity, (**b**) number distributions and (**c**) UV-Vis spectra of GaN-PEI, and GaN-PEI-Au at various gold concentrations.

To identify synthesis conditions that balance Au loading with dispersion stability, the HAuCl_4_·3H_2_O quantity was systematically varied (50–300 μL of 10 mM HAuCl_4_·3H_2_O solution) while keeping the GaN-PEI concentration constant. The resulting GaN-PEI-Au batches (GaN-PEI-Au-50-300) were evaluated by dynamic light scattering (DLS) and ζ-potential measurements (Figure 2a,b; Table 1), complemented by UV-Vis spectroscopy (Figure 2c).

The GaN-PEI purified dispersion exhibited a large hydrodynamic diameter (~826 nm) with a positive ζ-potential of +34.7 mV (Figure 2a,b; Table 1). The magnitude and sign of ζ-potential are consistent with a PEI-rich interface and electrostatic stabilization; however, the large DLS size suggests that the dispersion contains polymer-associated clusters and/or a small population of aggregates that dominate intensity-weighted DLS measurements. Upon addition of 50–100 μL of Au precursor, the apparent hydrodynamic diameter decreased to ~484–433 nm while ζ-potential remained near +34–+35 mV. At 150 μL of Au precursor, the lowest hydrodynamic diameter (~170 nm) was observed together with an increase in ζ-potential to +44.2 mV. Increasing the precursor further to 200–300 μL resulted in a modest increase in the hydrodynamic diameter to the ~200–300 nm range, with ζ-potential increasing to +55.8 mV.

Although adding inorganic Au mass is expected to increase particle size, the observed decrease in diameter at intermediate Au(III) amounts can be rationalized by considering that DLS reports a cooperative hydrodynamic size that includes the hydrated polymer corona and is highly sensitive to even minor aggregate fractions [26,27]. In polymer-coated colloids, changes in ionic strength and metal-polymer coordination can compact weak polyelectrolyte layers, effectively decreasing the thickness of the solvated PEI corona and reducing the apparent diffusion diameter measured by DLS [28,29]. In addition, Au nucleation within the PEI layer may reduce polymer bridging between neighboring particles (by “locking” segments into metal-polymer coordination environments), thereby decreasing clustering and lowering the aggregate contribution to the intensity-weighted size distribution [30]. These effects together can yield a smaller apparent hydrodynamic diameter even as Au nanoparticles are formed at the interface. At the highest Au(III) amounts, the size rebound is consistent with increasing heterogeneity and/or partial clustering promoted by elevated ionic strength, local Au-rich interparticle contacts, or broadened Au size distributions, effects that can occur even when ζ-potential remains strongly positive, because ζ-potential alone does not fully describe short-range attractions, patchy surfaces, or bridging-type interactions in polymer-metal hybrid colloids [31,32].

#### 2.2.2. Optical Evidence of Au Formation and Onset of Interparticle Coupling

UV-Vis spectra (Figure 2c) corroborate the formation of Au nanoparticles and provide insight into dispersion quality as a function of Au(III) amount. The GaN-PEI dispersion shows the characteristic weak near-UV absorption associated with GaN band-edge behavior (typically ~360–365 nm, depending on nanostructure and dispersion conditions) [33], which remains present across the GaN-PEI-Au series. After addition of HAuCl_4_·3H_2_O and thermal treatment, all GaN-PEI-Au samples exhibit a Au localized surface plasmon resonance (LSPR) band in the visible region (approximately 520–525 nm in aqueous media), confirming Au nanoparticle formation within the GaN-PEI system [34].

Importantly, the evolution of the LSPR band aligns with the colloidal trends from DLS/ζ-potential. At intermediate Au(III) amounts (50–200 μL), the LSPR remains near the green region with comparatively less broadening, consistent with well-dispersed Au nanoparticles and limited interparticle plasmon coupling. In contrast, at higher dosages (GaN-PEI-Au-250 and GaN-PEI-Au-300), the plasmon band broadens and shifts toward longer wavelengths, accompanied by the macroscopic color transition from pink/red to purple/blue (Figure 2c, inset). This spectral behavior is consistent with increased interparticle coupling and/or broader Au nanoparticle size distributions [35], both of which can emerge when Au loading becomes excessive and colloidal organization is partially lost [36].

DLS/ζ-potential and UV-Vis results indicated an improved dispersion and controlled Au nanoparticle formation at intermediate Au(III) amounts, followed by signs of increased coupling/heterogeneity at the highest dosages. The optimal condition should therefore be defined by the intended application: for electrochemical sensing, the best choice is typically the dosage that maintains dispersion stability and avoids pronounced plasmon broadening/red-shift (suggestive of aggregation/coupling) while maximizing electroactive Au nanoparticles coverage on GaN. Based on colloidal and optical data alone, the GaN-PEI-Au-200 sample represents the most favorable formulation, yielding the smallest apparent hydrodynamic diameter and maintaining high ζ-potential while still preserving a relatively narrow LSPR band.

#### 2.2.3. Morphological Analysis and EDX Elemental Analyses of GaN-PEI and GaN-PEI-Au Samples

**Figure 3:**
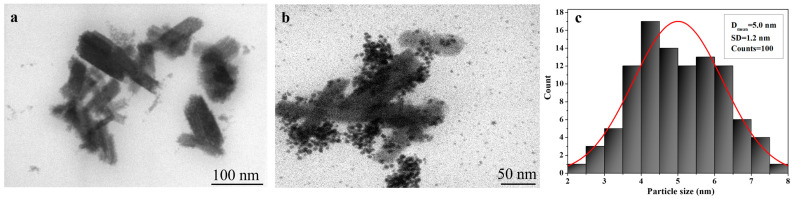
(**a**) STEM images of GaN-PEI; (**b**) GaN-PEI-Au-200; (**c**) particle size distribution histogram of Au nanoparticles decorating the GaN surface corresponding to GaN-PEI-Au-200 sample using ImageJ analysis.

STEM imaging supports the apparent differences between DLS and nanoscale morphology (Figure 3 and Figure S1). The GaN-PEI starting material displays GaN cores with an average diameter of approximately ~100 nm (Figure 3a and Figure S1a), consistent with particulate GaN supports coated by a hydrated PEI corona that contributes strongly to the diffusion-based DLS size.

In contrast, GaN-PEI-Au-200 clearly exhibits numerous high-contrast, quasi-spherical domains decorating the GaN surface (Figure 3b and Figure S1b–d), assigned to Au nanoparticles with a mean diameter of ~5 nm (Figure 3c). Importantly, this dimension represents the metallic Au nanoparticles and does not imply a reduction in the GaN core size: the GaN particles remain ~100 nm, while Au forms discrete nanoscale deposits anchored onto the PEI-functionalized interface [37]. Hence, the decrease in DLS-derived size upon metallization does not reflect “smaller particles,” but rather changes in the hydrated polymer layer and aggregate fraction, effects that can dominate the ensemble hydrodynamic diameter, even while Au is simultaneously deposited at the surface.

The STEM-derived Au nanoparticle size is also consistent with the UV-Vis response of GaN-PEI-Au-200. Quasi-spherical Au nanoparticles in the ~5 nm size range typically exhibit a characteristic LSPR band in the ~515–520 nm region in aqueous media; the comparatively narrow LSPR observed for GaN-PEI-Au-200 is therefore indicative of a relatively uniform Au population with limited interparticle plasmon coupling, in agreement with a well-dispersed composite architecture [38]. Furthermore, preferential decoration of specific surface sites or facets is commonly observed in PEI-mediated Au deposition, since amine-rich interfaces provide localized nucleation sites and can promote anisotropic spatial distribution of Au nanoparticles on crystalline substrates [39]. This behavior is consistent with the non-uniform but dense Au coverage visible on the faceted GaN surface in Figure 3b. In contrast, a higher Au(III) precursor concentration (GaN-PEI-Au-300) resulted in the formation of compact clusters, an increase in Au nanoparticle size to 13 nm, and partial nanoparticle detachment from the GaN surface (Figure S2), in agreement with the UV-Vis changes observed for the GaN-PEI-Au-250 and GaN-PEI-Au-300 samples.

**Figure 4:**
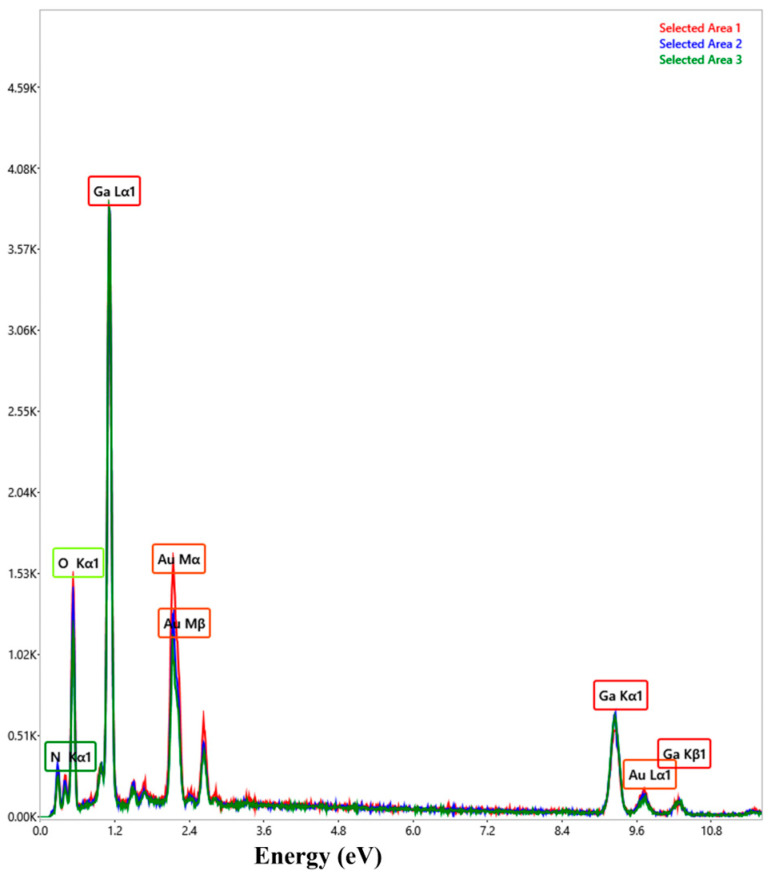
EDX spectra of GaN-PEI-Au-200 displaying characteristic peaks corresponding to Ga, Au, C, O, and N.

The EDX spectra of the GaN-PEI-Au-200 exhibited characteristic peaks corresponding to Ga, Au, C, O, and N (Figure 4). The signals associated with C and N confirm the presence of PEI within the nanocomposites, while the appearance of Au peaks indicates deposition and retention of gold on the GaN surface.

**Figure 5:**
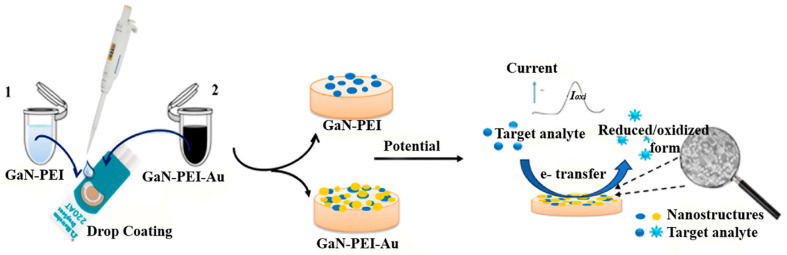
Schematic illustration of the drop-casting protocol for the deposition of GaN-PEI (1) and GaN-PEI-Au (2) films onto conventional gold electrodes.

Given their high colloidal stability, tunable surface chemistry, and controllable Au deposition, GaN-PEI and GaN-PEI-Au-200 samples were subsequently utilized as electrode modifiers for electrochemical sensing studies (Figure 5). Erythromycin was chosen as a representative analyte to evaluate how PEI functionalization and Au nanodeposition influence interfacial electron transfer, adsorption-driven preconcentration, and signal amplification on gold screen-printed electrodes.

### 2.3. Electrochemical Investigation of GaN-PEI-Au Nanocomposites

#### 2.3.1. Electrochemical Oxidation of Erythromycin at GaN-PEI and GaN-PEI-Au-Modified Electrodes

**Figure 6:**
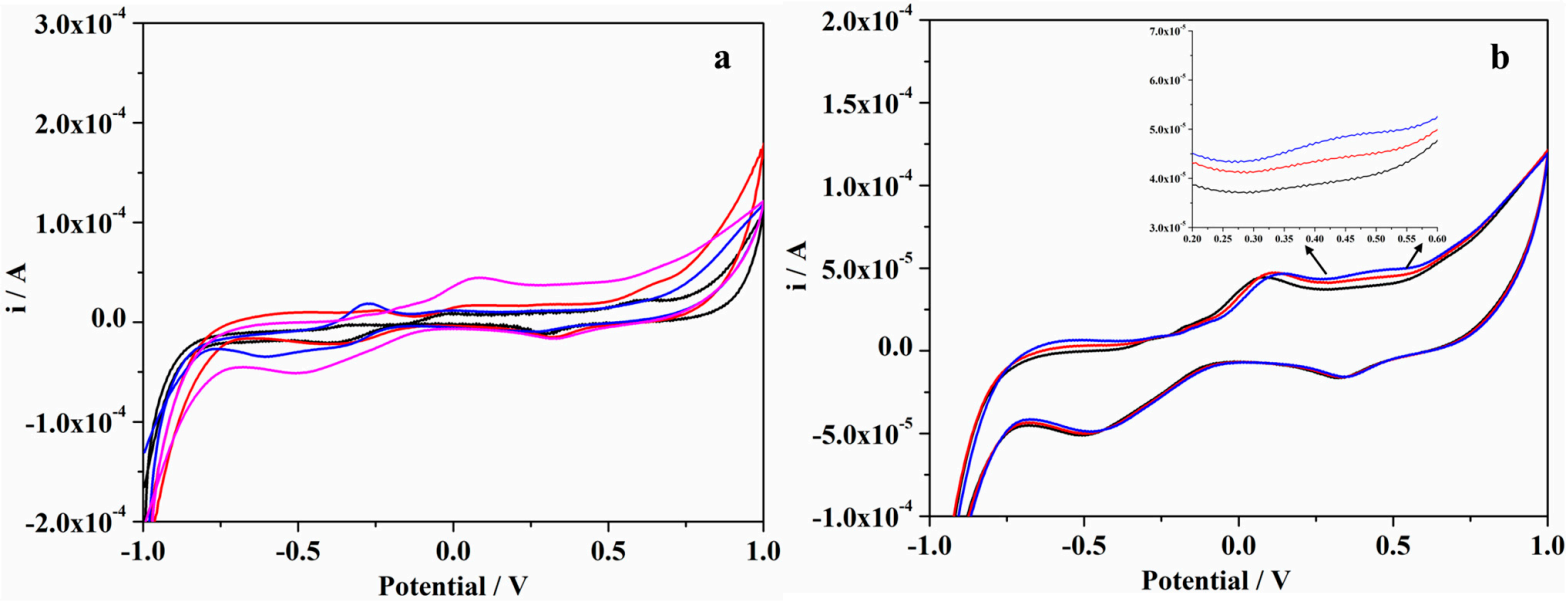
Cyclic voltammetry (CV) responses recorded in TAE buffer: (**a**) comparison between supporting electrolyte at the bare Au electrode (black) and the response to 2 mM erythromycin at bare Au (red), GaN-PEI-modified (blue), and GaN-PEI-Au-modified (purple) electrodes; (**b**) CV responses recorded at the GaN-PEI-Au-modified electrode upon successive additions of erythromycin: 2 mM (black), 4 mM (red), and 6 mM (blue).

The oxidation behavior of erythromycin was investigated using cyclic voltammetry (CV) and differential pulse voltammetry (DPV). The electrochemical oxidation behavior of erythromycin at the investigated electrode configurations was first examined using cyclic voltammetry (CV), which is widely employed as a preliminary electrochemical characterization technique to evaluate redox activity, electron-transfer behavior, and the qualitative response of electroactive analytes at modified electrodes. Comparative measurements were performed for the three electrode configurations (bare gold electrode, GaN-PEI and GaN-PEI-Au electrodes) in TAE buffer containing 2 mM erythromycin. The resulting voltammetric responses are presented in Figure 6a.

The CV measurements revealed a very weak anodic signal corresponding to the oxidation of erythromycin at approximately 0.2 V, which could be distinguished only at the GaN-PEI-Au-modified electrode. In contrast, the bare Au and GaN-PEI electrodes did not exhibit a clearly detectable oxidation peak under the same experimental conditions, indicating that the presence of Au nanoparticles within the GaN-PEI-Au nanocomposite facilitates the electron-transfer process and enhances the electrochemical response toward erythromycin. This observation is consistent with previously reported studies indicating that macrolide antibiotics, including erythromycin, typically produce weak voltammetric responses in CV due to their slow electron-transfer kinetics and the irreversible nature of the oxidation process. To further evaluate the concentration-dependent behavior, successive additions of erythromycin were performed while recording cyclic voltammograms at the GaN-PEI-Au electrode. As shown in Figure 6b, a gradual increase in the anodic peak current located around 0.2 V was observed with increasing erythromycin concentration, confirming that the recorded signal originates from the electrochemical oxidation of the analyte. Nevertheless, due to the relatively low current response obtained under cyclic voltammetric conditions, CV provided limited analytical sensitivity. Differential pulse voltammetry (DPV), which minimizes capacitive background currents and enhances faradaic signals, was therefore subsequently employed for the analytical investigations [40,41].

**Figure 7:**
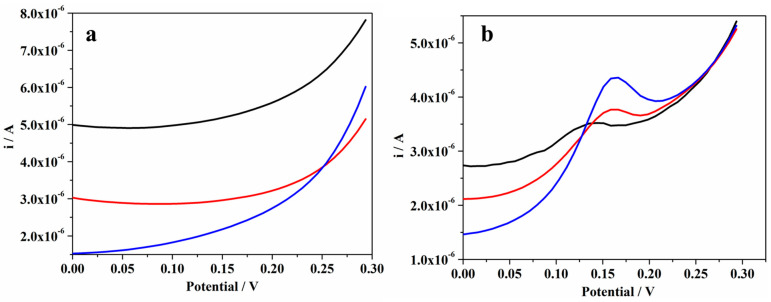
DPV responses recorded at bare Au (black), GaN–PEI-modified (red), and GaN–PEI–Au-modified (blue) electrodes in TAE buffer: (**a**) in the absence of erythromycin and (**b**) in the presence of 1 µM erythromycin.

Using DPV, comparative measurements were performed by recording voltammograms both in the absence (Figure 7a) and in the presence (Figure 7b) of erythromycin in TAE buffer. When only the supporting electrolyte was used, no oxidation peaks were detected within the explored potential window for any of the tested electrodes, confirming the lack of intrinsic redox activity and ensuring a stable, interference-free baseline. In contrast, the introduction of erythromycin produced well-defined oxidation peaks for all three electrode configurations within the potential range of 0.1–0.2 V, clearly indicating that the observed signals originate from the analyte’s electrochemical oxidation. The progressive increase in peak currents observed for Au, GaN-PEI, and GaN-PEI-Au electrodes indicates that the nanostructured GaN-PEI-Au interface not only facilitates more efficient electron transfer but also considerably enhances analytical sensitivity. This comparative approach clearly differentiates the analyte-specific faradaic response from the background behavior of the electrode materials.

The superior oxidation signal at GaN-PEI-Au electrodes is attributed to synergistic effects arising from the optimized nanocomposite architecture. Compared to bare gold electrodes, the GaN-PEI-modified surface benefits from the high electron mobility and nanostructured morphology of GaN, which facilitate charge transport and increase the number of accessible electroactive sites. In addition, the PEI layer promotes the adsorption of erythromycin through electrostatic interactions and improves film cohesion, leading to a more efficient interfacial electron transfer [42,43,44]. This effect of PEI is also supported by STEM (Figure 3a), which reveals a homogeneous dispersion of GaN particles induced by the presence of PEI on their surface [45]. The further decoration with Au nanoparticles produces the highest electrochemical response, as Au provides highly conductive electron-transfer pathways, increases the effective electroactive surface area, and lowers the charge-transfer resistance. Moreover, PEI ensures homogeneous Au nanoparticle immobilization, while Nafion enhances film stability and can locally preconcentrate the analyte. The synergy between the GaN-PEI support and Au nanoparticles thus accelerates heterogeneous electron transfer and maximizes faradaic currents, accounting for the superior performance of GaN-PEI-Au electrodes [12,46]. This is further confirmed by STEM analysis, which revealed discrete Au nanoparticles (~5 nm) anchored on ~100 nm GaN cores, a structure that provides a higher density of electroactive sites and more efficient electronic pathways compared with bare Au electrodes or GaN-PEI alone (Figure 3).

**Figure 8:**
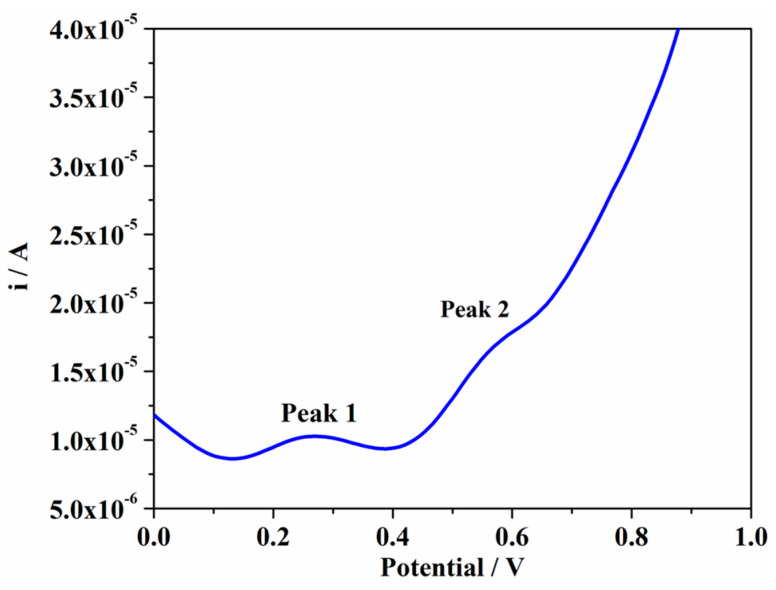
DPV response of erythromycin at the GaN–PEI–Au-modified electrode.

To further investigate the oxidation behavior of erythromycin and to confirm that its electrochemical mechanism is consistent with that reported in the literature [47,48], differential pulse voltammetry measurements were carried out over an extended potential window, as shown in Figure 8.

**Scheme 1:**
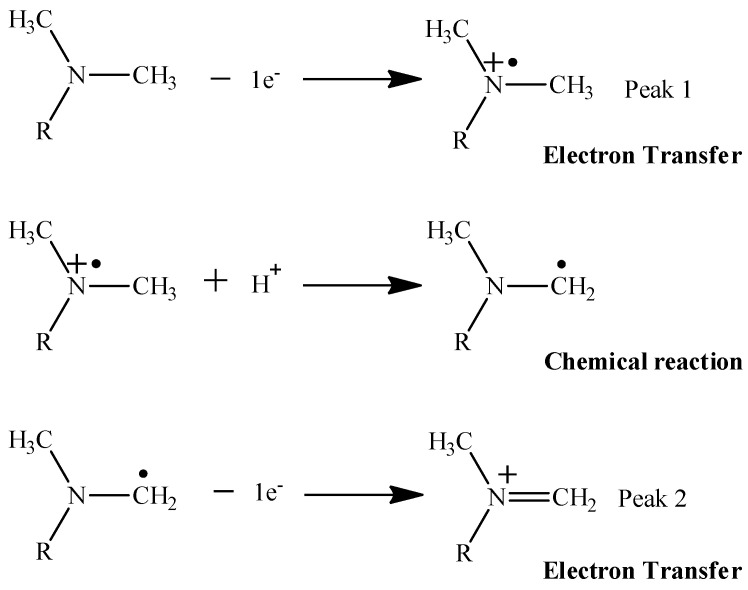
Pathway for electrochemical oxidation of erythromycin’s tertiary amine: aminium radical cation (Peak 1) to iminium ion (Peak 2).

Previously published electrochemical studies have shown that erythromycin exhibits two distinct oxidation peaks in voltammetric scans, which have been attributed to the sequential electrochemical oxidation of the tertiary amine moiety, as illustrated in Scheme 1. In particular, the first peak is associated with the one-electron removal from the protonated tertiary amine to form a radical cation, while the second peak corresponds to the further oxidation of the resulting intermediate species. The primary oxidation step involves a one-electron transfer from the protonated tertiary amine, leading to the formation of a radical cation. This process is irreversible and appears at relatively low anodic potentials, consistent with the oxidation behavior of tertiary amines reported in the literature. In voltammetric measurements, this step gives rise to a well-defined anodic peak and is attributed to the initial electron-transfer reaction of erythromycin. Following the formation of the radical cation, the molecule becomes electrochemically unstable and undergoes subsequent chemical transformations, including bond cleavage and structural degradation of the macrolide ring. These follow-up reactions can be further oxidized at higher potentials, resulting in a secondary anodic peak associated with oxidative degradation products of erythromycin [47,48,49].

In the obtained DPV profiles, two well-defined anodic peaks were also observed over the expanded potential window, consistent with these mechanistic descriptions. The presence of these two peaks confirms that the electrochemical oxidation of erythromycin under the current experimental conditions followed the same mechanistic pathway described in the literature.

#### 2.3.2. Influence of the Supporting Electrolyte on the Electrochemical Oxidation of Erythromycin

To study the effect of the supporting electrolyte on erythromycin’s electrochemical response, DPV measurements were performed in various media, including TAE buffer, PBS, H_2_SO_4_, acetic acid, and KOH (Figure S3). The results show that the electrochemical signal strongly depends on both the electrolyte composition and pH. Only TAE buffer produced a well-defined and reproducible oxidation peak at ~0.2 V, while PBS, acidic, and strongly alkaline media yielded weak or indistinct signals. This behavior aligns with erythromycin’s oxidation mechanism, involving its tertiary amine. In acidic media, full protonation stabilizes the molecule and shifts oxidation to higher potentials, reducing the signal. Strongly alkaline media can also lower signal intensity due to hydrolysis and macrolide ring instability. TAE buffer, with a moderately alkaline pH (~8), partially protonates the tertiary amine, allowing efficient electron transfer while maintaining molecular stability and adsorption, resulting in a clear and reproducible peak. TAE buffer was thus selected as the optimal supporting electrolyte for further electrochemical studies [50].

#### 2.3.3. Calibration

**Figure 9:**
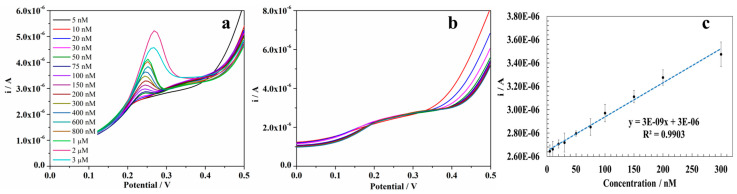
(**a**) Differential pulse voltammograms recorded at the GaN-PEI-Au electrode upon successive additions of erythromycin for calibration purposes. (**b**) Assessment of the electrochemical stability of the GaN-PEI-Au electrode by ten consecutive DPV scans recorded in TAE buffer prior to analyte addition. (**c**) Calibration plot of erythromycin on the GaN-PEI-Au electrode.

In order to evaluate the analytical performance of the GaN-PEI-Au-modified electrode for erythromycin determination, a DPV calibration was carried out. The measurements were performed in TAE buffer within the potential range from −0.3 to +0.5 V, using successive additions of erythromycin into the electrochemical cell under stirring. The erythromycin concentration was progressively increased from 5 nM to 3 µM, and a DPV voltammogram was recorded after each addition. Figure 9a displays the DPV responses recorded at the GaN-PEI-Au electrode upon successive additions of erythromycin. With increasing analyte concentration, the anodic peak current associated with erythromycin oxidation increases systematically, while the peak potential remains nearly constant, indicating a stable and reproducible electrochemical process. Prior to the addition of erythromycin, the electrochemical stability of the modified electrode was evaluated, as shown in Figure 9b, by performing ten consecutive DPV scans in the supporting electrolyte (TAE buffer) in the absence of erythromycin. The negligible variation in current response confirms the good stability of the GaN-PEI-Au electrode and its suitability for reliable quantitative measurements.

The resulting calibration plot (Figure 9c) showed a linear response over the 5 nM to 2 µM range, with a correlation coefficient of 0.990, confirming reproducible, concentration-dependent oxidation of erythromycin. From the calibration data, the electrode sensitivity was determined to be 1.32 × 10^−3^ µA/nM, and the limit of detection was 52.5 nM. These results highlight the GaN-PEI-Au electrode’s capability for sensitive and reliable erythromycin detection at nanomolar concentrations. The obtained results are consistent with previously reported data on the electrochemical detection of erythromycin (Table 2).

## 3. Materials and Methods

### 3.1. Chemicals and Reagents

All chemicals were of analytical grade and were used as received without further purification. Gallium nitride nanoparticles (GaN, 99.9% trace metals basis), erythromycin and branched polyethyleneimine (bPEI, MW ≈ 25 kDa), tris-acetate-EDTA buffer (TAE buffer, 50× stock, pH 8.3), gold(III) chloride trihydrate (HAuCl_4_·3H_2_O, 99.9% trace metals basis), and Nafion™ perfluorinated resin solution were purchased from Sigma-Aldrich GmbH (Steinheim, Germany). Ultrapure water (18.2 MΩ·cm, Milli-Q grade) was produced using a TKA GenPure system (model 08.2204) and used throughout.

### 3.2. Instruments and Measurements

UV-Vis absorption spectra of GaN-PEI and GaN-PEI-Au dispersions were recorded using a Lambda 35 UV-Vis spectrophotometer (Perkin Elmer, Waltham, MA, USA) in quartz cuvettes (10 mm optical path length). Measurements were performed in aqueous dispersions; ultrapure water was used as the reference blank in double-beam mode.

Hydrodynamic size and ζ-potential were determined with a Malvern ProRED Particle Size Analyzer (Malvern Panalytical, Malvern, UK). Disposable plastic cuvettes (DTS0012; 10 × 10 mm, Malvern Panalytical, Malvern, UK) were used for dynamic light scattering (DLS), while disposable folded capillary cells (DTS1070, Malvern Panalytical, Malvern, UK) were used for ζ-potential measurements. Data were processed using ZS XPLORER software (version 3.3.0.42, Malvern Panalytical, Malvern, UK).

Scanning transmission electron microscopy (STEM) images were acquired using a Verios G4 UC scanning electron microscope (Thermo Scientific, Prague, Czech Republic) operating in STEM mode (STEM 3+ detector, 30 kV). Samples were prepared by depositing 6 μL of GaN-PEI or GaN-PEI-Au dispersion onto 400-mesh carbon-coated copper grids (Ted Pella, Redding, CA, USA) and drying in a dust-free environment at room temperature (23 °C) for 24 h. The size of the gold nanoparticles was determined from STEM images using ImageJ software (version 1.48r, National Institutes of Health, Bethesda, MD, USA). The images were first calibrated using the scale bar provided in the micrographs. The diameters of individual Au nanoparticles were then measured, and a total of 100 particles were analyzed to determine the average particle size and size distribution.

Energy-dispersive X-ray spectroscopy (EDX) elemental analysis was performed in three different areas using a Thermo Scientific Verios G4 UC scanning electron microscope equipped with an energy-dispersive X-ray spectroscopy analyzer (Octane Elect Super SDD detector, EDAX, Mahwah, NJ, USA). For this analysis, the samples were drop-cast onto aluminum stubs and allowed to dry at room temperature prior to measurement.

Electrochemical measurements were conducted using a PalmSens potentiostat (Palm Instrument BV, Houten, The Netherlands) controlled by the manufacturer’s software. Cyclic voltammetry (CV) and differential pulse voltammetry (DPV) were used to evaluate the electrochemical response of ERY at bare and nanomaterial-modified electrodes. Measurements were performed in TAE buffer using a three-electrode screen-printed electrode (SPE) platform (Metrohm DropSens, Oviedo, Spain) with a planar configuration: a gold working electrode (4 mm diameter), a gold counter electrode, and a silver reference electrode (Ag). CV was employed to investigate the electrochemical behavior of ERY over a potential window from −1.0 V to +1.0 V (vs. Ag reference). DPV parameters were: modulation time 50 ms, pulse amplitude 100 mV, and step potential 8 mV, over a potential window from −0.30 V to +0.50 V (vs. Ag reference).

### 3.3. Preparation of GaN-PEI-Au

#### 3.3.1. GaN-PEI Synthesis

GaN-PEI conjugates were prepared by dispersing 22 mg of GaN nanoparticles in 4 mL of bPEI solution (1% *w*/*v*). The suspension was stirred at 400 rpm and 45 °C for 3 h to promote surface functionalization and obtain a homogeneous dispersion. The mixture was then allowed to equilibrate at room temperature for 24 h. Purification was performed by centrifugal ultrafiltration (Amicon Ultra-15, 100 kDa molecular-weight cutoff, Merck, Darmstadt, Germany) to remove excess/unbound PEI and low-molecular-weight impurities. The dispersion was centrifuged at 10,000× *g* (RCF) for 10 min, followed by two washing cycles with ultrapure water (2 × 1.0 mL). The purified GaN–PEI conjugates were re-dispersed in ultrapure water and adjusted to a final volume of 4 mL.

#### 3.3.2. GaN-PEI-Au Optimization

Gold deposition on GaN-PEI was optimized by varying the gold precursor amount while maintaining a constant GaN-PEI concentration (5.5 mg/mL). Aliquots (1.0 mL) of GaN-PEI dispersion were transferred into six Eppendorf tubes and incubated in a thermomixer at 90 °C under agitation (600 rpm). When the dispersions reached approximately 80 °C, aqueous HAuCl_4_·3H_2_O (10 mM) was added to each tube (50, 100, 150, 200, 250, or 300 μL). After 2 h, the dispersions exhibited distinct color changes (pink/red to purple/blue), indicative of Au nanostructure formation. Samples were stored at 4 °C in the dark until further characterization.

#### 3.3.3. Preparation of the Modified Electrodes

GaN-PEI and GaN-PEI-Au dispersions (5.5 mg/mL) were each mixed with isopropanol at a 1:1 (*v*/*v*) ratio. Nafion™ (final 2% solution, as prepared from the commercial stock) was added as a binder to improve film adhesion and mechanical stability on the electrode surface. The resulting dispersions had a final nanomaterial concentration of 2.75 mg/mL and were ultrasonicated for 30 min to ensure homogeneity. For electrode modification, 3.0 μL of each dispersion was drop-cast onto the gold working electrode surface and allowed to dry at room temperature to form a uniform nanomaterial layer (Figure 1).

## 4. Conclusions

In this work, we developed a hybrid nanostructured electrode modifier based on polyethyleneimine-functionalized gallium nitride decorated with gold nanoparticles (GaN-PEI-Au) for enhanced electrochemical sensing. PEI served as a multifunctional interfacial layer that stabilized GaN dispersions, introduced amine-rich surface functionality, and enabled controlled in situ formation of Au nanoparticles at the GaN interface. Systematic variation in the Au precursor concentration demonstrated that gold loading strongly influences colloidal organization and optical response, with intermediate Au(III) volumes yielding the most favorable balance between dispersion stability and controlled Au nanoparticle formation, as supported by DLS/ζ-potential and UV-Vis spectroscopy.

Morphological characterization by STEM confirmed that GaN-PEI-Au consists of ~100 nm GaN cores decorated with discrete Au nanoparticles (~5 nm), providing a high-density conductive surface architecture without compromising particle integrity. When implemented as an electrode coating, the GaN-PEI-Au interface produced a pronounced amplification of the erythromycin oxidation signal compared to bare Au and GaN-PEI-modified electrodes, consistent with synergistic contributions from the nanostructured GaN scaffold, the PEI-mediated interfacial stabilization, and the Au nanoparticle network facilitating electron transfer and increasing the electroactive surface area. The erythromycin oxidation response followed the mechanistic behavior reported in the literature, with two anodic peaks observed over an extended potential range, supporting a sequential oxidation pathway involving the tertiary amine functionality.

Under optimized DPV conditions, the GaN-PEI-Au-modified electrode enabled quantitative erythromycin determination with a linear response from 5 nM to 2 µM (R^2^ = 0.990), a sensitivity of 1.32 × 10^−3^ µA nM^−1^, and a limit of detection of 52.5 nM, while exhibiting stable baseline behavior during repeated scans. These findings highlight the potential of GaN-PEI-Au hybrid nanocomposites as robust and tunable platforms for electrochemical sensing. The modular design strategy introduced here can be extended to other analytes by tailoring the interfacial chemistry and nanostructure composition, supporting future development of sensitive sensing interfaces for pharmaceuticals and related contaminants in complex matrices.

## Figures and Tables

**Table 1:** Hydrodynamic diameter (Z-average), polydispersity index (PDI), and ζ-potential of GaN-PEI and GaN-PEI-Au at various gold concentrations measured by dynamic light scattering. Each sample was analyzed in triplicate and results are presented as mean ± standard deviation.

Sample	Z-Average (nm)	PDI	ζ-Potential (mV)
GaN-PEI-Au50	484.2 ± 14.3	0.34 ± 0.02	34.0 ± 0.2
GaN-PEI-Au100	432.2 ± 4.2	0.32 ± 0.04	35.4 ± 0.4
GaN-PEI-Au150	169.6 ± 5.8	0.39 ± 0.01	44.2 ± 1.0
GaN-PEI-Au200	162.3 ± 1.4	0.36 ± 0.02	43.1 ± 0.5
GaN-PEI-Au250	221.2 ± 0.9	0.32 ± 0.03	52.1 ± 0.2
GaN-PEI-Au300	141.3 ± 0.4	0.30 ± 0.02	55.8 ± 0.1
GaN-PEI	826.3 ± 22.8	0.27 ± 0.02	34.7 ± 0.7

**Table 2:** Comparison of literature-reported data and present results for the electrochemical detection of erythromycin.

Electrode Modification	Detection Technique	LOD	Linear Range	Sensitivity	Ref.
screen-printed carbon electrodes with sodium dodecyl sulfate surfactant	Amperometry	0.14 mg L^−1^ (0.19 µM)	0.14–15 mg L^−1^	-	[51]
screen-printed carbon electrodes with molecularly imprinted polymers	Differential Pulse Voltammetry	1.9 × 10^−8^ mol L^−1^	5.0 × 10^−8^–1.0 × 10^−5^ mol L^−1^	-	[52]
gold electrode with CS-Pt and GR-Au nanocomposites + MIP	Cyclic Voltammetry/Amperometry	2.3 × 10^−8^ mol L^−1^	7.0 × 10^−8^–9.0 × 10^−5^ mol L^−1^	-	[53]
glassy carbon electrode with acetylene black nanoparticle-modified	Cyclic Voltammetry	8 × 10^−8^ mol L^−1^	2 × 10^−7^–1 × 10^−5^ mol L^−1^	-	[47]
glassy carbon electrode with arginine molecularly imprinted polymer	Differential Pulse Voltammetry/ Cyclic Voltammetry	2.0 × 10^−9^ mol L^−1^	3 × 10^−9^–1.6 × 10^−6^ mol L^−1^	57,300 µA cm^−2^ µM^−1^	[54]
screen-printed electrode with molecularly imprinted polymer based on m-phenylenediamine	Differential Pulse Voltammetry	1 × 10^−10^ mol L^−1^	-	-	[55]
glassy carbon electrode with carbon nanohorns, polydopamine and erythromycin molecularly imprinted membrane	Cyclic Voltammetry/Differential Pulse Voltammetry	2.7 × 10^−9^ mol L^−1^	1.0 × 10^−5^–1.0 × 10^−8^ mol L^−1^	-	[56]
carbon electrode with erythromycin molecularly imprinted polymer and multiwalled carbon nanotubes and polyaniline	Potentiometric	9.6 ± 0.4 × 10^−7^ mol L^−1^	4.6 × 10^−6^ to 1.0 × 10^−3^ M	-	[57]
GaN-PEI-Au-modified gold electrode	Differential Pulse Voltammetry	52.5 nM	5 nM–2 µM	1.32 × 10^−3^ µA nM^−1^	This work

## Data Availability

All data generated or analyzed during this study are included in this published article and its Supplementary Materials.

## Supplementary Materials

The following supporting information can be downloaded at: https://www.mdpi.com/article/10.3390/ijms27062728/s1.
