# Supplementary material for: Polyethyleneimine-Directed In Situ Gold Deposition on Gallium Nitride Nanoparticles for Enhanced Electrochemical Detection of Erythromycin
Source: Int J Mol Sci. 2026 Mar 17;27(6):2728. doi: 10.3390/ijms27062728 (PMC13026566; doi:10.3390/ijms27062728)
Supplement: Supplementary file 1 [file ijms-27-02728-s001.zip › ijms-4176815-supplementary.pdf]

Polyethyleneimine-Directed *In Situ* Gold Deposition on Gallium Nitride Nanoparticles for Enhanced Electrochemical Detection of Erythromycin

Oana Elena Carp, Denisse-Iulia Bostiog, Elena Laura Ursu, Rares-Georgian Mocanu, Narcisa Laura Marangoci Ion Tiginyanu, Alexandru Rotaru

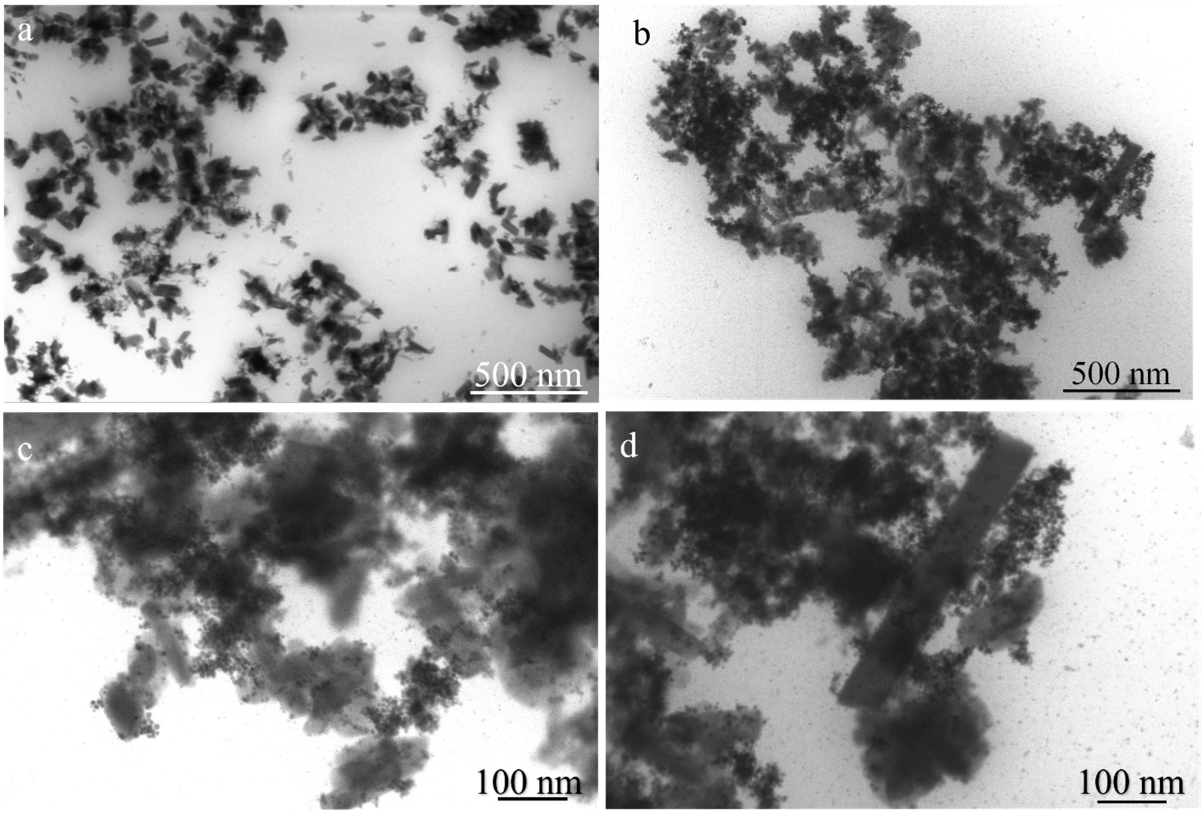

Figure S1. Representative STEM micrographs of (a, b) GaN–PEI and (c, d) GaN–PEI–Au nanocomposites, showing Au nanoparticle deposition on the PEI-functionalized GaN surface.

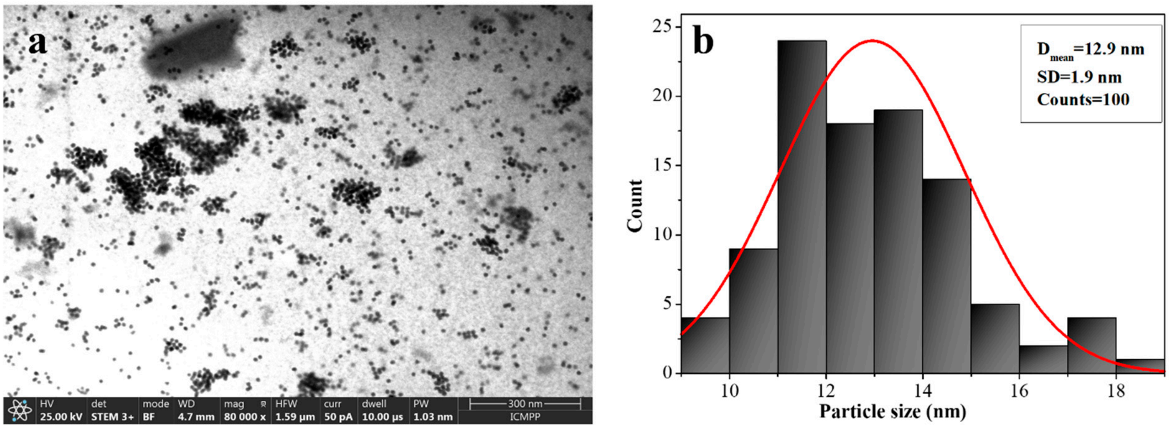

Figure S2. STEM images of GaN-PEI-Au-300 (a); particle size distribution histogram of Au nanoparticles using ImageJ analysis (b).

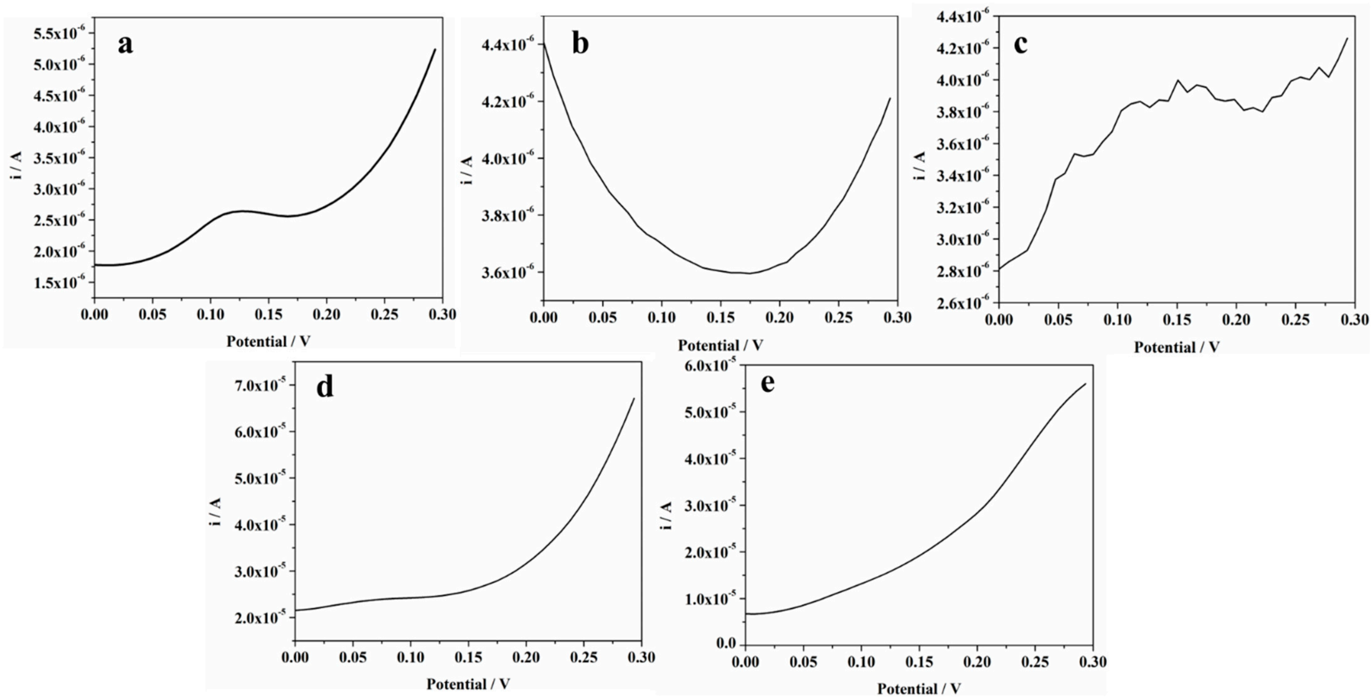

Figure S3. Differential pulse voltammograms of 1 μM erythromycin in various supporting electrolytes: (a) TAE buffer, (b) PBS, (c) H_2SO_4, (d) acetic acid (AA), and (e) KOH. Measurements highlight the influence of electrolyte composition and pH on the oxidation peak at ~0.2 V.
